# Using circulating O-sulfotyrosine in the differential diagnosis of acute kidney injury and chronic kidney disease

**DOI:** 10.1186/s12882-021-02268-3

**Published:** 2021-02-23

**Authors:** Shuai Chen, Yong-Hua Liu, Dao-Peng Dai, Zheng-Bin Zhu, Yang Dai, Zhi-Ming Wu, Li-Ping Zhang, Zhi-Feng Duan, Lin Lu, Feng-Hua Ding, Jin-Zhou Zhu, Rui-Yan Zhang

**Affiliations:** 1 Department of Vascular & Cardiology, Rui Jin Hospital, Shanghai Jiao Tong University School of Medicine, Shanghai, China; 2 Department of Cardiology, Bao Shan People’s Hospital, Baoshan, Yunnan Province China

**Keywords:** Acute kidney injury, Chronic kidney disease, Metabolite, Tyrosine O-sulfation, O-sulfotyrosine, Renal dysfunction

## Abstract

**Background:**

Sulfation of tyrosine, yielding O-sulfotyrosine, is a common but fixed post-translational modification in eukaryotes. Patients with increased circulating O-sulfotyrosine levels experience a faster decline in renal function with progression to end-stage renal disease (ESRD). In the present study, we measured serum O-sulfotyrosine levels in individuals with chronic kidney disease (CKD) and acute kidney injury (AKI) to explore its ability to differentiate AKI from CKD.

**Methods:**

A total of 135 patients (20 with AKI and 115 with CKD) were recruited prospectively for liquid chromatography-mass spectrometry assessment of circulating O-sulfotyrosine. We also studied C57BL/6 mice with CKD after 5/6 nephrectomy (Nx). Blood samples were drawn from the tail vein on Day 1, 3, 5, 7, 14, 30, 60, and 90 after CKD. Serum separation and characterization of creatinine, blood urea nitrogen (BUN), and O-sulfotyrosine was performed. Thus, the time-concentration curves of the O-sulfotyrosine level demonstrate the variation of kidney dysfunction.

**Results:**

The serum levels of O-sulfotyrosine were markedly increased in patients with CKD compared with AKI. Median O-sulfotyrosine levels in CKD patients versus AKI, respectively, were as follows:243.61 ng/mL(interquartile range [IQR] = 171.90–553.86) versus 126.55 ng/mL (IQR = 48.19–185.03, *P* = 0.004). In patients with CKD, O-sulfotyrosine levels were positively correlated with creatinine, BUN, and Cystatin C (r = 0.63, *P* < 0.001; r = 0.49, *P* < 0.001; r = 0.61, *P* < 0.001, respectively) by the multivariate linear regression analysis (β = 0.71, *P* < 0.001; β = 0.40, *P* = 0.002; β = 0.73, *P* < 0.001, respectively). However, this association was not statistically significant in patients with AKI (r = − 0.17, *P* = 0.472; r = 0.11, *P* = 0.655; r = 0.09, *P* = 0.716, respectively). The receiver operating characteristic (ROC) analysis illustrated that the area under the curve was 0.80 (95% confidence interval [CI] 0.71–0.89; *P* < 0.001) and the optimal cut-off value of serum O-sulfotyrosine suggesting AKI was < 147.40 ng/mL with a sensitivity and specificity of 80.90 and 70.00% respectively. In animal experiments, serum levels of O-sulfotyrosine in mice were elevated on Day 7 after 5/6 nephrectomy (14.89 ± 1.05 vs. 8.88 ± 2.62 ng/mL, *P* < 0.001) until Day 90 (32.65 ± 5.59 vs. 8.88 ± 2.62 ng/mL, *P* < 0.001).

**Conclusion:**

Serum O-sulfotyrosine levels were observed correlated with degrading renal function and in CKD patients substantially higher than those in AKI patients. Thus serum O-sulfotyrosine facilitated the differential diagnosis of AKI from CKD.

## Introduction

Chronic kidney disease (CKD) is defined as structural or functional kidney damage that lasts longer than three months and adversely affects multiple metabolic pathways [1]. Acute kidney injury (AKI), however, is a sudden episode of kidney failure or kidney damage that happens within a few hours or days and may be accompanied by the retention of nitrogenous wastes in the body [2]. CKD and AKI commonly develop with mutual symptoms in the medical history, pathological data, ultrasonographic findings and biopsy, making differential diagnosis essential to the clinical. It is particularly challenging for physicians to diagnose during the initial admission for uremia patients who present an unknown kidney dysfunction history.

Metabolites are small organic molecules that are involved in a variety of biological processes. The kidneys produce and secrete metabolites [3]. Changes in blood metabolite concentration may be due to impaired renal function because of the changes in generation, filtration, secretion, reabsorption, or metabolism. Tyrosine sulfation, yielding O-sulfotyrosine, is a common post-translational modification of the membrane and secreted proteins [2, 4, 5]. This post-translational modification effectively transfers sulfate from 3′-phosphate 5′-phosphosulfate to a tyrosine residue within a peptide chain, which is catalyzed by a family of membrane-bound tyrosyl protein sulfotransferases (TPST) [3, 6, 7]. Data suggest that tyrosine sulfation plays an essential role in regulating protein-protein interactions and protein transport. Previous studies showed that protein tyrosine O-sulfation is implicated in several biological processes such as hemostasis, inflammation response, and chemokine receptor recognition [8, 9]. The modification is found in various conditions, including HIV infection, atherosclerosis, hemostasis, and inflammatory response [4, 10–15].

Recent studies showed that elevated levels of O-sulfotyrosine are associated with disease progression in CKD patients. Indeed, individuals with increased circulating O-sulfotyrosine levels experienced faster renal function decline, leading to end-stage renal disease (ESRD) [3, 7]. However, there are relatively few studies assessing O-sulfotyrosine in patients with AKI. In the present study, we measured the serum O-sulfotyrosine levels in individuals with AKI and CKD to explore its potential in differentiating AKI from CKD.

## Methods

### Study population

A total of 150 patients referred to the Department of Cardiology of Rui Jin Hospital, Shanghai Jiao Tong University School of Medicine, with glomerular filtration rates (GFR) < 60 mL/min 1.73 m^2^ were recruited in the study. Fifteen patients with type 1 diabetes, chronic viral or bacterial infections, tumor, and immune disorders were excluded. And to avoid the interference of inflammation on the results, we did not enroll patients with nephritis. Thus, the remaining 135 patients were enrolled in the final analyses. CKD was manifested as either a GFR less than 60 mL/min per 1.73 m2, the kidney damage markers, or both, that persisted for at least three months [1, 16, 17]. And AKI was diagnosed with any of the followings: increase in creatinine by ≥0.3 mg/dL (≥26.5 μmol/L) within 48 h; increase in creatinine to ≥1.5 times the baseline, which was known or presumed to have occurred within the previous seven days; or urine volume < 0.5 mL/kg/h for six hours [16, 18]. And the majority of AKI patients had transient renal failure after cardiac surgery. Finally, twenty and one hundred and fifteen patients were diagnosed with AKI and CKD, respectively.

The study protocol was approved by the Institutional Review Board of Rui Jin Hospital, Shanghai Jiaotong University School of Medicine. The clinical investigation was conducted per the principles of the Declaration of Helsinki. Written informed consent was obtained from all recruited patients.

### Animal experiments and 5/6 nephrectomy model of CKD

All animal experiments were conducted per the Guide for the Care and Use of Laboratory Animals by the US National Institutes of Health (NIH Publication No.85–23, revised 1996) and approved by the Animal Care Committee of Shanghai Jiaotong University School of Medicine. 8–10 weeks old male C57BL/6 mice were selected, and in-house bred in a pathogen-free environment at the Animal Experiment Center of Rui Jin Hospital, Shanghai Jiaotong University School of Medicine. The animals were allowed access to food and water ad libitum on a 12-h light/dark cycle.

Subtotal nephrectomy (5/6 Nx) was performed on the mice as described to establish CKD [19]. In brief, C57BL/6 mice (*n* = 6) were placed on a heated table to maintain average body temperature, then anesthetized with pentobarbital. The left kidney was exposed and 2/3 surgically removed. After surgery, each mice was fed with a regular diet and administered with three-days penicillin intraperitoneal injection (200,000 U per day). And the sham-operated animals were also given penicillin injections for 3 days. One week after the nephrectomy, the renal vessels and ureters of the right kidney was exposed, which were ligated with cotton thread, then the kidney tissue between the hilum and the ligated part was cut. Meanwhile, the sham-operated animals underwent kidney exposure only.

Serum BUN and creatinine levels were measured weekly by an automatic biochemical analyzer (BX3010, Sysmex). The mice were considered CKD if serum creatinine values were twice than those of the sham group [19, 20]. Additionally, the serum levels of O-sulfotyrosine in mice were measured on Day 1, 3, 5, 7, 14, 30, 60, and 90 after the operation, respectively.

### Liquid chromatography-mass spectrometry determination of serum O-sulfotyrosine

Serum levels of O-sulfotyrosine were analyzed using a high-performance liquid chromatography system (1260 series, Agilent Technologies; Palo Alto, CA, USA) and mass spectrometer (Agilent 6460, Agilent Technologies; Palo Alto, CA, USA) as previously described [21, 22]. Patient and mouse serum were collected, and 1.5 mL of chloroform/methanol (2:1, v/v) was added. The solution was vortexed for one minute and then centrifuged for 10 min at 3000 g. Next, an 800 μL organic phase adding into a cleaning tube, dried with nitrogen. Mass spectrometry analysis was conducted by adding 200 μL of isopropanol/methanol solution (1:1, v:v), and the supernatant was transferred to HPLC vials for LC-MS analysis. A Waters UPLC BEH C18 column (2.1 mm × 50 mm, 1.7 μm, Waters, USA) was used for chromatographic separation. Multiple reaction monitoring mode was used to detect O-sulfotyrosine. The analysis was performed by Shanghai Applied Protein Technology Inc.

### Biochemical studies

Blood samples were obtained from all patients after an overnight fast. Samples were stored at − 80 °C before analysis. We use the same serum sample for O-sulfotyrosine detection and biochemical studies. Serum levels of creatinine, BUN, triglyceride, total cholesterol, low-density lipoprotein (LDL) cholesterol, high-density lipoprotein (HDL) cholesterol, glucose were assessed (HITACHI 912 Analyzer, Roche Diagnostics, Germany). Blood HbA1c concentration was measured using ion-exchange high performance liquid chromatography with Bio-rad Variant Hemoglobin Testing System (Bio-Rad Laboratories, USA). GFR was estimated using the Chronic Kidney Disease Epidemiology Collaboration (CKD-EPI) equation: GFR_EPI_ (mL/min/1.73 m^2^) = 141 × min (creatinine/k, 1)^α^ × max (creatinine/k, 1)^− 1.209^ × 0.993^age^ × 1.018 [if female], where k is 0.7 for females and 0.9 for males, α is - 0.329 for women and − 0.411 for men, min indicates the minimum of creatinine/k or 1, and max indicates the maximum of creatinine/k or 1 [23].

### Statistical analysis

Data are presented as mean ± standard deviation (SD) or median (interquartile range) for continuous variables and number (percentage) for categorical variables. The differences between groups were evaluated by Student t tests and Chi-square test for continuous and categorical variables, respectively. Pearson’s and Spearman’s correlation tests were adopted to assess the relationship between variables. Receiver operating characteristic (ROC) analyses were used to determine the power of O-sulfotyrosine concentration for detecting the presence of AKI. We performed a one-way analysis of variance for animal experiments to assess the significance of changes relative to the controls. All calculations used two-sided tests with an overall significance level of alpha = 0.05. SPSS 25.0 for Windows (SPSS, Inc., Chicago, IL, USA) in the statistical analyses.

## Results

### Demographics of the study population

The baseline characteristics and parameters of both CKD and AKI patients are described in detail in Table 1. There were no statistically significant differences in age, gender, body mass index, history of hypertension between individuals with AKI and CKD. However, those with CKD had more coronary artery disease (*P* < 0.001) but were less likely to smoke than those with AKI (*P* = 0.006). Biochemical tests showed that serum Cystatin C levels were more elevated (*P* = 0.002), and BUN was lower in patients with CKD (*P* = 0.005). There were no significant differences in creatinine, GFR, calcium, HbA1c, total cholesterol, triglyceride, or left ventricular ejection fraction between the two groups.

**Table 1:** Baseline characteristics in patients with chronic kidney disease and acute kidney injury

Characteristics	Chronic kidney disease (***n*** = 115)	Acute kidney injury (***n*** = 20)	***P value***
Male, n (%)	77 (67.0)	11 (55.0)	0.300
Age, year	68.33 ± 11.11	66.10 ± 8.50	0.395
Body mass index, kg/m^2^	24.36 ± 3.64	24.34 ± 3.34	0.986
Smoking, n (%)	13 (11.3)	7 (35.0)	0.006
Diabetes mellitus, n (%)	37 (32.2)	7 (35.0)	0.803
Hypertension, n (%)	93 (80.9)	14 (70.0)	0.268
Dyslipidemia, n (%)	6 (5.2)	1 (5.0)	0.968
Coronary artery disease, n (%)	100 (87.0)	7 (35.0)	< 0.001
Serum BUN, mmol/L	12.95 ± 7.51	18.22 ± 8.16	0.005
Serum creatinine, μmol/L	285.80 ± 294.73	199.20 ± 104.77	0.197
Serum uric acid, μmol/L	437.27 ± 105.86	443.70 ± 148.96	0.815
GFR, mL/min/1.73m^2^	34.86 ± 20.64	33.42 ± 16.72	0.768
Calcium, mmol/L	2.22 ± 0.17	2.25 ± 0.15	0.407
Phosphorus, mmol/L	1.28 ± 0.35	1.14 ± 0.24	0.079
Total cholesterol, mmol/L	4.02 ± 1.19	4.19 ± 1.56	0.583
Triglyceride, mmol/L	1.64 ± 1.42	1.46 ± 0.72	0.597
HDL cholesterol, mmol/L	1.06 ± 0.26	1.14 ± 0.38	0.236
LDL cholesterol, mmol/L	2.39 ± 0.91	2.57 ± 1.15	0.444
HbA1c, %	6.29 ± 1.06	6.42 ± 1.17	0.645
Cystatin C, mg/L	2.92 ± 2.23	1.30 ± 0.33	0.002
LVEF, %	60.74 ± 10.68	60.55 ± 12.38	0.943

Values are given as mean ± standard deviation (SD) or number (percentage)

*BUN* blood urea nitrogen, *GFR* glomerular filtration rate, *HbA1c* glycated hemoglobin, *HDL* high-density lipoprotein, *LDL* low-density lipoprotein, *LVEF* left ventricular ejection fraction

### O-sulfotyrosine is significantly increased in individuals with CKD compared to those in AKI

**Fig. 1:**
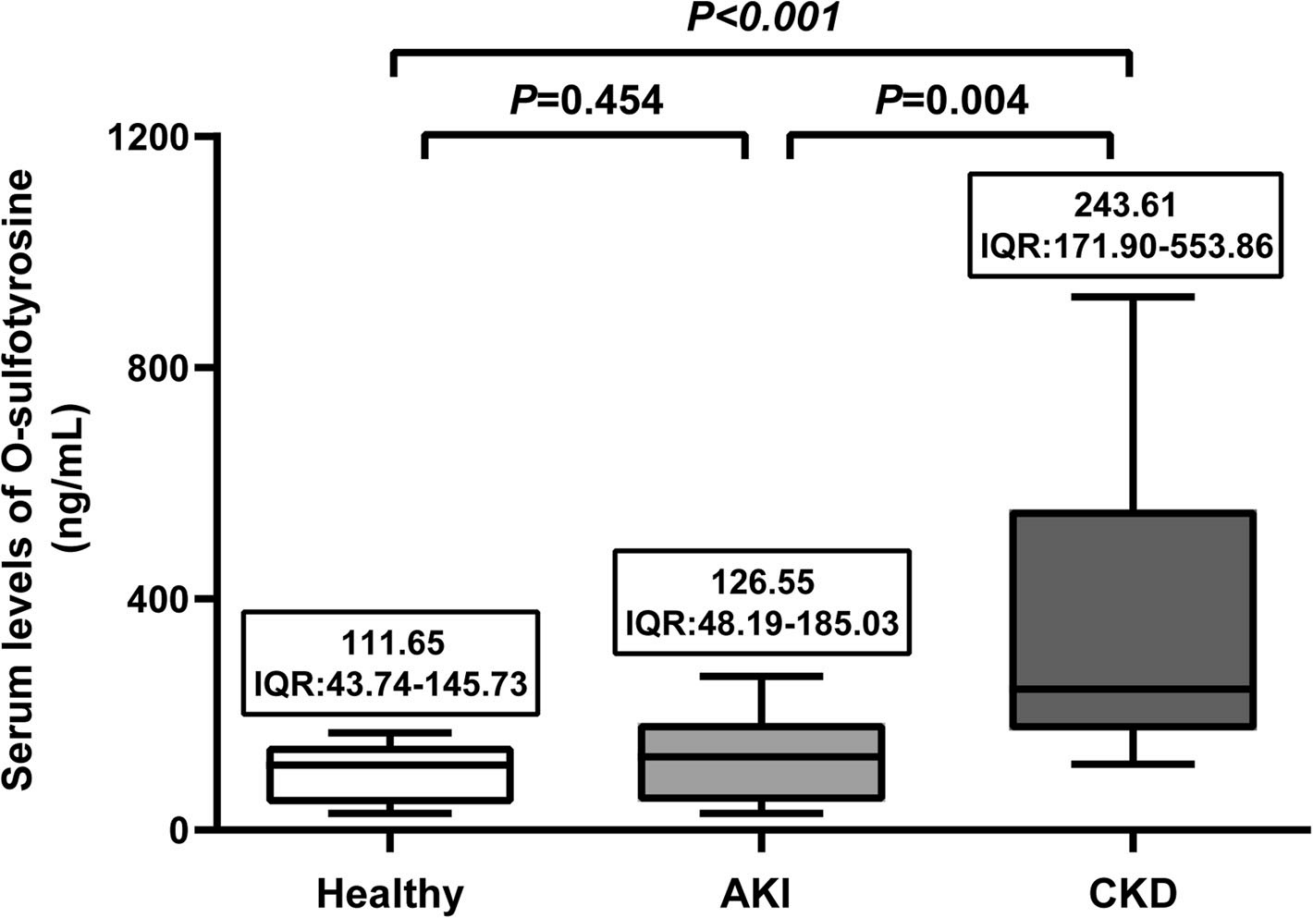
Comparison of serum O-sulfotyrosine levels between healthy volunteers and individuals with CKD and AKI. AKI, acute kidney injury; CKD, chronic kidney disease; IQR, interquartile range

**Fig. 2:**
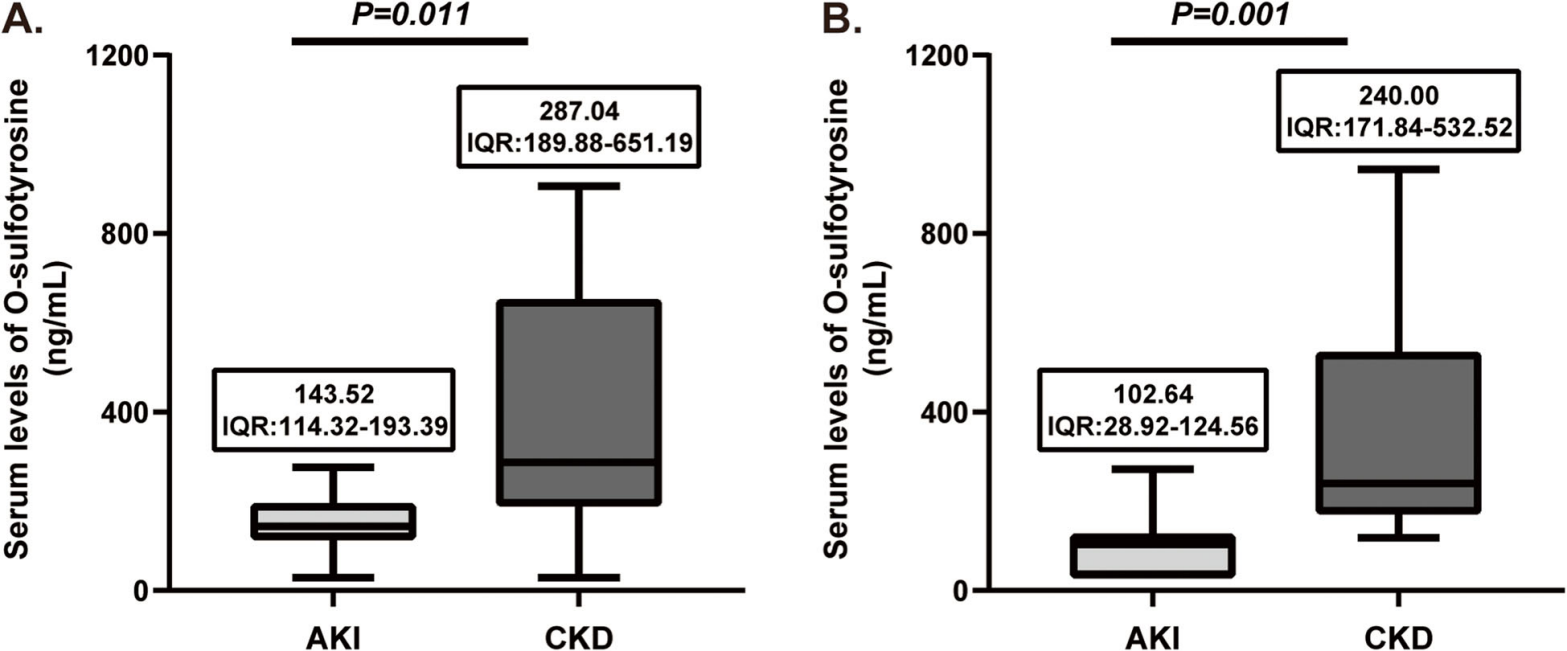
Comparison of serum levels of O-sulfotyrosine in patients with or without coronary artery disease. Comparison of serum O-sulfotyrosine levels between individuals with CKD and AKI in all populations without coronary artery disease (**a**). Comparison of serum O-sulfotyrosine levels between individuals with CKD and AKI in all populations with coronary artery disease (**b**). AKI, acute kidney injury; CKD, chronic kidney disease; IQR, interquartile range

Serum levels of O-sulfotyrosine were markedly increased in individuals with CKD compared with those with AKI (243.61, interquartile range [IQR] 171.90–553.86 ng/mL vs. 126.55, IQR 48.19–185.03 ng/mL; *P* = 0.004) (Fig. 1). We have also measured the serum level of O-sulfotyrosine in healthy volunteers. We found that there was no significant difference between AKI patients and healthy volunteers (126.55 ng/mL, IQR:48.19–185.03 vs. 111.65 ng/mL, IQR:43.74–168.02, *P* = 0.454). However, serum levels of O-sulfotyrosine were markedly increased in individuals with CKD compared with healthy volunteers (243.61 ng/mL, IQR: 171.90–553.86 vs. 111.65 ng/mL, IQR: 43.74–168.02, *P* < 0.001) (Fig. 1). In addition, we compared serum O-sulfotyrosine levels in those with CKD and AKI combined with coronary artery disease or without coronary artery disease. We found that serum O-sulfotyrosine levels in those with CKD were higher than those with AKI, both with and without coronary artery disease (Fig. 2).

**Fig. 3:**
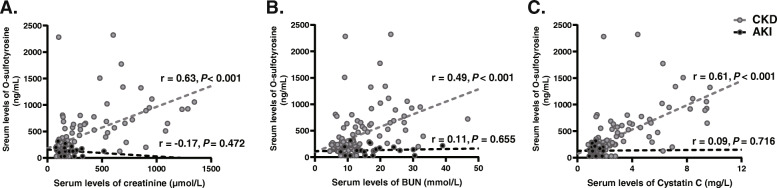
Correlation of O-sulfotyrosine with creatinine (**a**), BUN(**b**), and Cystatin C(**c**) in individuals with CKD and AKI. Gray circle and dashed line, CKD; black circle and dashed line, AKI. BUN, blood urea nitrogen; CKD, chronic kidney disease; AKI, acute kidney injury

**Fig. 4:**
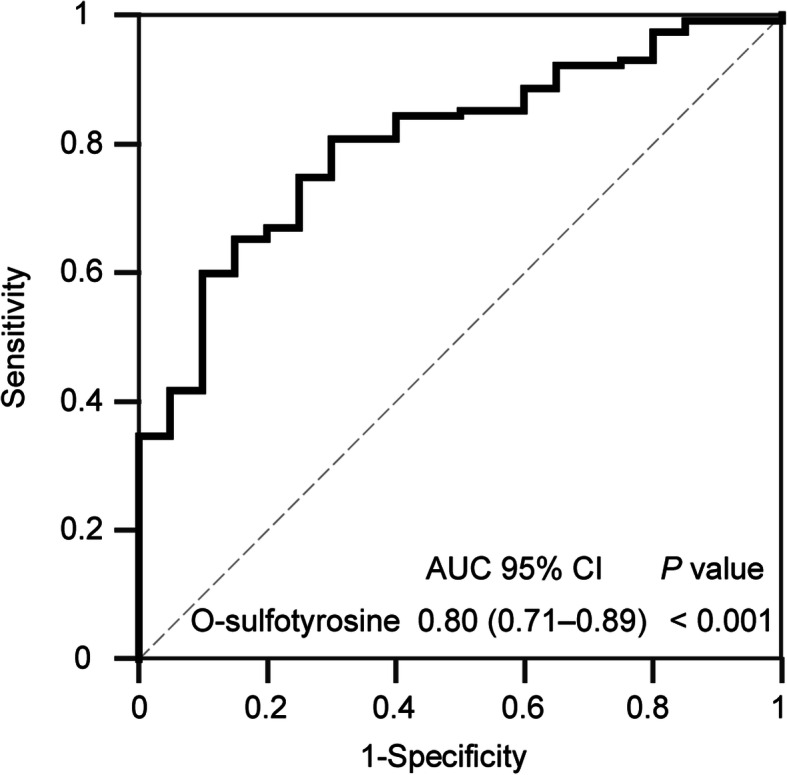
Receiver operating characteristic curve analysis for detecting AKI. Area under the curve was 0.80 (95% CI, 0.71–0.89; *P* < 0.001) and the optimal cut-off value of serum O-sulfotyrosine suggesting AKI was < 147.40 ng/mL with a sensitivity and specificity of 80.90 and 70.00% respectively

In CKD patients, O-sulfotyrosine levels were positively correlated with creatinine, BUN, and Cystatin C (r = 0.63, *P* < 0.001; r = 0.49, *P* < 0.001; r = 0.61, *P* < 0.001, respectively) (Fig. 3). After adjustment for confounding factors (age, gender, body mass index, diabetes mellitus, calcium, and phosphorus), creatinine, BUN, and Cystatin C remained correlated significantly with O-sulfotyrosine levels in multivariate linear regression analysis (β = 0.71, *P* < 0.001; β = 0.40, *P* = 0.002; β = 0.73, *P* < 0.001, respectively). However, this association was not evident in AKI patients (r = − 0.17, *P* = 0.472; r = 0.11, *P* = 0.655; r = 0.09, *P* = 0.716, respectively). The receiver operating characteristic (ROC) analysis showed that the area under the curve was 0.80 (95% confidence interval [CI] 0.71–0.89; *P* < 0.001) and the optimal cut-off value of serum O-sulfotyrosine in detecting AKI was < 147.40 ng/mL with a sensitivity and specificity of 80.90 and 70.00%, respectively (Fig. 4).

### Serum levels of O-sulfotyrosine in mice gradually increase after 5/6 Nx

**Fig. 5:**
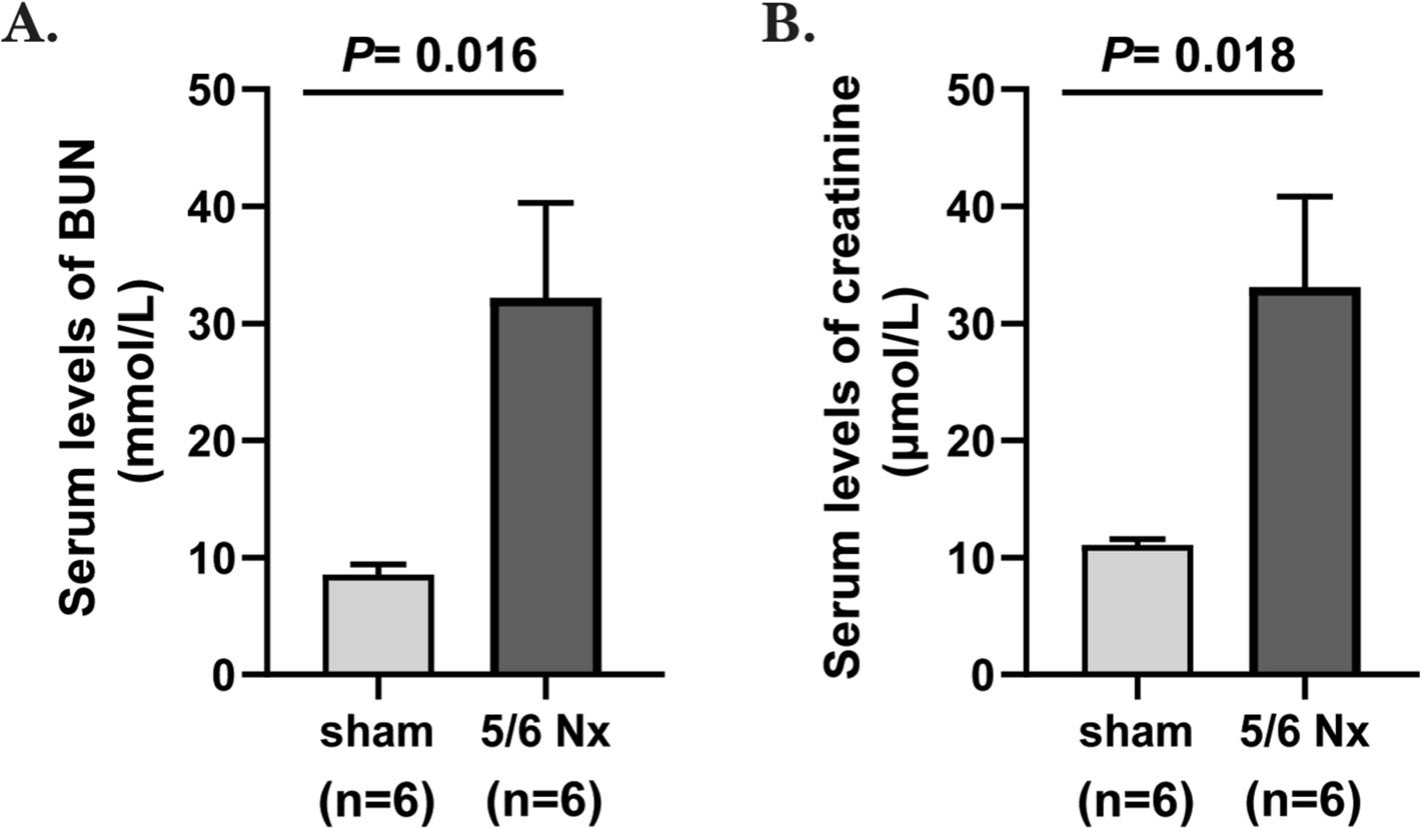
Serum levels of BUN (**a**), and creatinine (**b**) in mice 7 days after 5/6 Nx. BUN, blood urea nitrogen

**Fig. 6:**
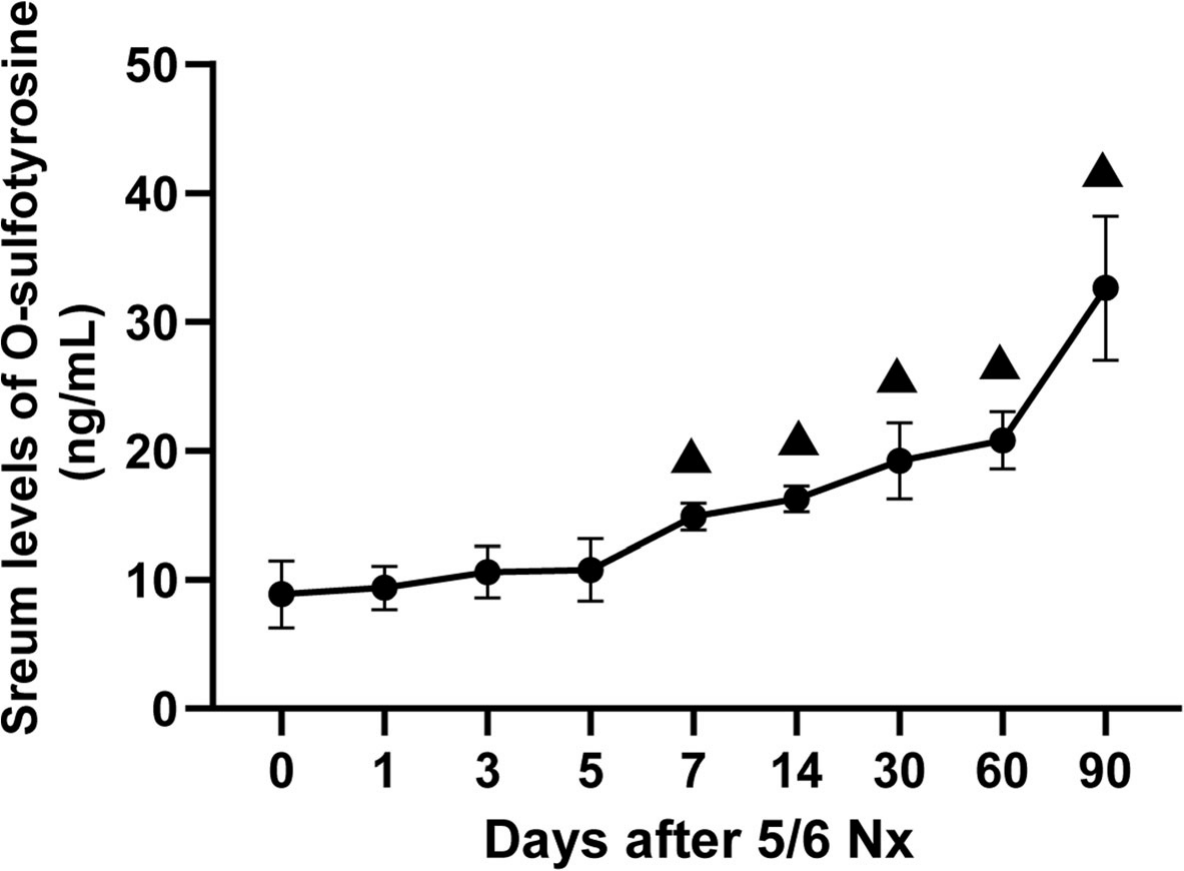
The serum levels of O-sulfotyrosine in mice at different time points after 5/6 Nx. **▲** *P* < 0.05 versus the serum levels of O-sulfotyrosine before operation

To verify the clinical findings, the 5/6 nephrectomy model was established in mice to observe the change in the trend of O-sulfotyrosine over time after kidney dysfunction. Kidney function was substantially impaired in animals subjected to 5/6 Nx. And the serum levels of BUN and creatinine in mice were measured weekly after surgery to confirm CKD’s presence on mice. The serum levels of BUN (32.20 ± 19.90 vs. 8.55 ± 2.14 mmol/L, *P* = 0.016) and creatinine (33.09 ± 19.01 vs. 11.08 ± 1.24 μmol/L, *P* = 0.018) 7 days after the 5/6 Nx group were higher than those before surgery (Fig. 5a-b). Serum levels of O-sulfotyrosine in mice were increasingly elevated on day 7 after the 5/6 nephrectomy (14.89 ± 1.05 vs. 8.88 ± 2.62 ng/mL, *P* < 0.001) until 90 days (32.65 ± 5.59 vs. 8.88 ± 2.62 ng/mL, *P* < 0.001) (Fig. 6).

## Discussion

The present study demonstrates that the serum levels of O-sulfotyrosine were significantly increased in patients with CKD relative to those with AKI. Interestingly, the serum levels of creatinine, BUN, and Cystatin C in CKD patients were significantly correlated with their O-sulfotyrosine levels. In rodents, the serum O-sulfotyrosine levels gradually increased from day 7 after CKD establishment. Statistically, the indication of O-sulfotyrosine < 147.40 ng/mL could be adopted to differentiate AKI from CKD with an 80.90% sensitivity and 70.00% specificity. These results provide clinical insight for physicians to make the differential diagnosis.

Tyrosine sulfation is a common post-translational modification, and the metabolite is called O-sulfotyrosine. This modification is found in various conditions, including HIV infection, atherosclerosis, hemostasis, and inflammatory response. But the role of tyrosine sulfation modification in renal pathophysiology has not been thoroughly studied. Previous studies show that elevated O-sulfotyrosine is associated with the progression of kidney disease. The KORA F4 study [3] employed a nontargeted metabolomics approach using gas and liquid chromatography coupled with mass spectrometry to assess whether metabolism levels are related to the reduction of GFR. It found that O-sulfotyrosine was significantly associated with annual GFR decline. And higher serum levels of O-sulfotyrosine were associated with more massive GFR decline and incident CKD. Additionally, in a prospective cohort study involving participants with type 1 diabetes, proteinuria, and impaired baseline renal function, patients with elevated O-sulfotyrosine levels in the circulating blood had a faster decline in renal function leading to end-stage renal disease [7]. The accumulation of O-sulfotyrosine in the body may be due to impaired kidney function and decreased kidney ability to process O-sulfotyrosine. However, AKI is kidney failure or kidney damage that occurs suddenly within a few hours or days. It is unclear whether the impairment of kidney function in the AKI setting will cause O-sulfotyrosine accumulation.

In the present study, we found that elevated levels of O-sulfotyrosine were associated with decreased renal function in patients with CKD but not AKI. This was very interesting, suggesting that there may be complex reasons for the increase in O-sulfotyrosine in those with CKD other than a decrease in the glomerular filtration rate. Protein-tyrosine O-sulfation effectively transfers sulfate from 3′-phosphate 5′-phosphosulfate to a tyrosine residue within a peptide chain, which is catalyzed by a family of membrane-bound tyrosyl protein sulfotransferases (TPST-1 & TPST-2) [3, 6, 7]. Inorganic sulfate retention invariably occurs in patients with renal function insufficiency, as documented by well-known increases in its plasma levels. Increased levels of sulfate in the plasma can increase the formation of sulphur-containing cofactors such as “active sulphate (adenosine 3’-phosphate 5’-sulphatophosphate)” [24], which eventually results in an increase in tyrosine sulfation modification. As this process may be relatively time-dependent, it is not surprising that CKD was associated with more significant O-sulfotyrosine increases. This indicated that more O-sulfotyrosine is metabolized in patients with CKD. In general, serum O-sulfotyrosine levels in CKD patients were higher than those in individuals with AKI, mainly because of the increased metabolism of O-sulfotyrosine in individuals with CKD and a decreased glomerular filtration rate, which leads to increased retention.

To further observe the change trend of O-sulfotyrosine over time after kidney dysfunction, 5/6 nephrectomy model was established in mice. As predicted, animals with CKD post-nephrectomy also had increased serum levels of O-sulfotyrosine, which continued to increase with time. Serum levels of O-sulfotyrosine in mice were increasingly elevated on day 7 after the 5/6 nephrectomy until 90 days. And the O-sulfotyrosine increase delay suggests that serum levels of O-sulfotyrosine are time-dependent.

Many studies have recently explored improvements in AKI’s differential diagnosis from CKD with high sensitivity and specificity [25–28]. However, few of these studies found a time-dependent relationship between these biomarkers and kidney injury. So, it is not fully verified that these biomarkers can be used to describe the severity and duration of renal disease. In our study, we found O-sulfotyrosine was positively associated with decreaed renal function in patients with CKD but not AKI. And O-sulfotyrosine levels differed significantly from those of control mice starting at Day 7 after 5/6 nephrectomy and increased throughout the 90 days of evolution. Thus, our experiments in patients and animals demonstrate that elevated O-sulfotyrosine levels are associated with chronic kidney injury. O-sulfotyrosine can be used as a marker to identify AKI from CKD.

There are several limitations to our study. First, this is a cross-sectional study allowing for the detection of associations instead of causality. Second, the overall sample size was modest. Also, the majority of AKI patients had transient renal failure after cardiac surgery. In contrast, most of the individuals with CKD had hypertensive, diabetic, or nephritic nephropathy. Thus it impacted the ability to evaluate the association between O-sulfotyrosine and other chronic diseases. Moreover, other biomarkers also could be used to discriminaite AKI and CKD. It would be helpful if we test these makers and compared them with O-sulfotyrosine. Unfortunately, these experimants can’t be made up in the present study.

## Conclusion

In summary, this study indicates that serum O-sulfotyrosine made aid in distinguishing AKI from CKD. Additional prospective multi-center studies are needed to elucidate better the pathophysiological significance of increased circulating O-sulfotyrosine in kidney disease.

## Data Availability

The datasets used and/or analyzed during this study are available from the corresponding author on reasonable request.

## Abbreviations

AKI: Acute kidney injury
BUN: Blood urea nitrogen
CKD: Chronic kidney disease
CKD-EPI: Chronic kidney disease epidemiology collaboration
CI: Confidence interval
ESRD: End-stage renal disease
GFR: Glomerular filtration rates
HbA1c: Glycated hemoglobin
HDL: High-density lipoprotein cholesterol
IQR: Interquartile range
LDL: Low-density lipoprotein cholesterol
LVEF: Left ventricular ejection fraction
Nx: Nephrectomy
ROC: Receiver operating characteristic
TPST: Tyrosylprotein sulfotransferases
